# MicrobTiSDA: A flexible R package for inferring interspecies interactions and abundance dynamics in microbiome time‐series data

**DOI:** 10.1002/imo2.70067

**Published:** 2025-11-27

**Authors:** Shijia Li, Remco Kort, Tim G. J. de Meij, Stanley Brul, Meike T. Wortel, Johan A. Westerhuis

**Affiliations:** ^1^ Molecular Biology & Microbial Food Safety, Swammerdam Institute for Life Sciences University of Amsterdam Amsterdam The Netherlands; ^2^ Biosystem Data Analysis, Swammerdam Institute for Life Sciences University of Amsterdam Amsterdam The Netherlands; ^3^ Amsterdam Institute for Life and Environment (A‐LIFE) Vrije Universiteit Amsterdam Amsterdam The Netherlands; ^4^ Amsterdam Gastroenterology Endocrinology Metabolism Research Institute Amsterdam UMC Amsterdam The Netherlands

## Abstract

*MicrobTiSDA* is a user‐friendly and flexible R package specifically designed for longitudinal microbiome data analysis. By integrating process‐oriented functional modules—including data input, data preprocessing, interspecies interaction inference, natural spline regression modeling, temporal pattern clustering, and random forest classification—*MicrobTiSDA* enables users to efficiently and systematically infer reliable interspecies interactions and accurately characterize microbial abundance dynamics from time‐series data. The package is compatible with diverse experimental designs and provides comprehensive, intuitive visualizations, thereby substantially enhancing both the efficiency of longitudinal microbiome data analysis and the depth of biological interpretation.

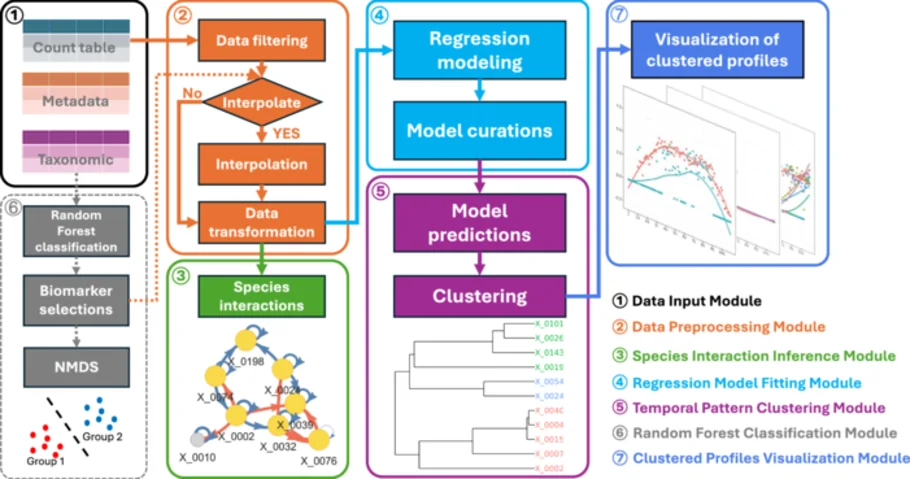

To the editor,

With the rapid advancement of high‐throughput sequencing technologies, longitudinal microbiome studies have become increasingly important for elucidating the temporal dynamics of host‐associated and environmental microbial communities. Unlike cross‐sectional studies that provide only static snapshots, longitudinal approaches capture abundance trajectories, community stability, and responses to external interventions, thereby offering unique perspectives on the dynamic characteristics of microbial ecosystems [1, 2, 3, 4, 5]. However, efficient and reliable tools that can simultaneously characterize temporal changes in microbial abundance and uncover the ecological interactions shaping community dynamics remain limited.

To address this gap, we developed *MicrobTiSDA*, an R package that integrates regression‐based modeling with the discrete‐time Lotka‐Volterra (dLV) framework. This tool enables users to infer interspecies interactions, identify keystone species, analyze species abundance trajectories, and detect microbial clusters with coherent temporal patterns. We demonstrated the utility and effectiveness of *MicrobTiSDA* by applying it to three longitudinal datasets: in vitro aquatic microbiomes, infant gut microbiomes, and preterm infant gut microbiomes associated with sepsis.

By bridging regression approaches with interaction modeling, *MicrobTiSDA* provides a flexible, user‐friendly, and comprehensive toolkit for advancing longitudinal microbiome research. This package offers robust support for elucidating underlying ecological mechanisms.

## 1 OVERVIEW OF *MICROBTISDA* WORKFLOW

*MicrobTiSDA* is an R package specifically designed for the analysis of microbiome time‐series data. It integrates seven core functional modules: data input, data preprocessing, interspecies interaction inference, temporal regression modeling of species abundance, temporal pattern clustering, random forest classification, and visualization (Methods detailed in Supporting Information). Users begin by providing a standardized species count table (samples as rows, microbial features as columns), accompanied by sample metadata and taxonomic annotation. The preprocessing module supports filtering of microbial features based on minimum total abundance and prevalence across samples, interpolation of missing time points to construct continuous time series, and modified centered log‐ratio (MCLR) transformation. To capture ecological dynamics, *MicrobTiSDA* incorporates the “*Learning Interactions from MIcrobial Time Series*” (LIMITS) algorithm [6]—based on the discrete‐time Lotka‐Volterra (dLV) model [7]—to infer interspecies interaction coefficients, and employs natural spline regression to model abundance trajectories over time. Based on the regression results, users can cluster microbial features according to similarity in temporal patterns and visualize the outcomes through built‐in plotting functions. Furthermore, for studies with group‐based experimental designs, *MicrobTiSDA* includes a random forest classification module to identify microbial features that are discriminatory across conditions.

## 2 EVALUATION OF NATURAL SPLINE REGRESSION MODEL PERFORMANCE

The modeling of microbial feature abundances using natural spline regression constitutes one of the core functionalities of *MicrobTiSDA*. To systematically evaluate its performance, we designed a benchmarking study based on 10‐fold cross‐validation. We utilized the aquatic microbiome in vitro culture data set reported by Fujita et al. [8], focusing on the eight independent replicates cultivated with peptone medium. For each replicate, regression models were constructed to capture the temporal dynamics of all microbial features across 110 consecutive days of observations, ensuring continuity and completeness of the time series.

We compared the natural regression implementation in *MicrobTiSDA* against three widely used time‐series modeling methods: stepwise polynomial regression (*maSigPro*) [9], spline‐based regression (*MetaDprof*) [10], and locally weighted regression (*LOWESS*) from the *MetaLonDA* package [11]. Model performance was evaluated using multiple metrics derived from a comparison of predicted and observed values, including Root Mean Squared Error (RMSE), Mean Absolute Error (MAE), Mean Absolute Percentage Error (MAPE), Coefficient of Determination (*R* ^2^), and Bias. Wilcoxon rank‐sum tests were applied to assess the significance of performance differences between methods.

The results indicated that *MicrobTiSDA* achieves comparable or superior performance across most metrics (Figure 1). Notably, in terms of MAE, the natural spline regression model significantly outperformed *maSigPro*'s stepwise polynomial regression in most replicates (except Rep.2), underscoring its advantages in prediction accuracy and robustness. Although no significant differences were observed across the other four metrics overall, *MicrobTiSDA* yielded significantly lower RMSE values than *maSigPro* in the first and sixth replicates (Rep.1 and Rep.6), further supporting its improved error control under specific conditions.

**Figure 1:**
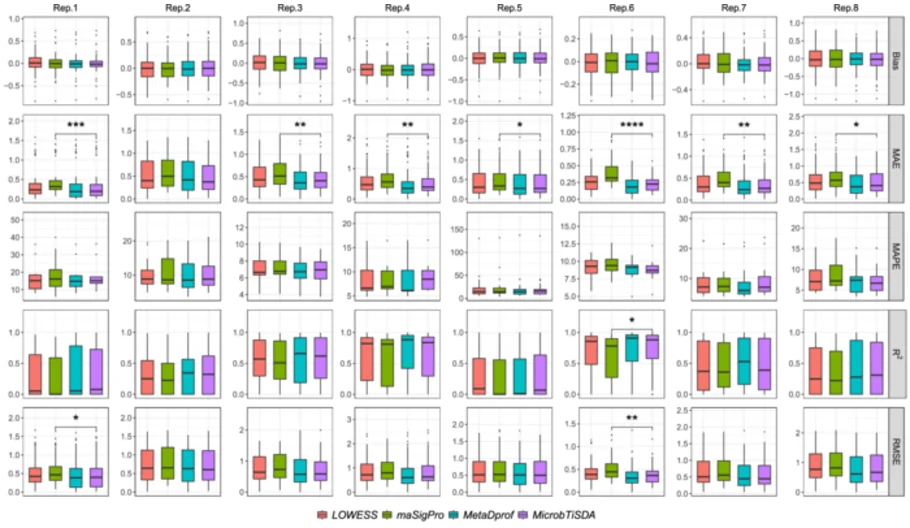
Boxplot comparison of model performance across all microbial features in eight aquatic microbiome replicates, evaluated using root mean squared error (RMSE), mean absolute error (MAE), mean absolute percentage error (MAPE), coefficient of determination (*R* ^2^), and Bias. *MicrobTiSDA* was compared against *MetaDprof*, *maSigPro*, and *LOWESS*. Rep.1 to Rep.8 denote eight independent replicates. Statistical significance of performance differences was assessed using Wilcoxon rank‐sum tests (*p < 0.05; **p < 0.01; ***p < 0.005; ****p < 0.001).

Collectively, these findings demonstrate that the natural spline regression model in *MicrobTiSDA* performs robustly in modeling microbial time‐series abundances, with particular strength in minimizing mean absolute error compared to conventional polynomial regression approaches. These results affirm the utility and reliability of *MicrobTiSDA* for characterizing temporal dynamics in microbial feature abundances.

## 3 EVALUATION OF INTERSPECIES INTERACTION INFERENCE PERFORMANCE

Another core functionality of *MicrobTiSDA* is its capability to infer interspecies interactions from microbiome time‐series data. To evaluate this functionality, we simulated a test data set with known interactions using the dLV model (detailed in Supporting Information). To test the dLV estimation procedure of the interspecies interactions, the known interspecies interaction matrix *C*_*n*_ = {*c*_*nij*_} was randomly generated: off‐diagonal interaction coefficients were sampled from a normal distribution N(0, 0.05^2^), while diagonal entries (self‐inhibition terms) were set as negative values $-\left|N(0.1,0.05^{2})\right|$. The initial abundance *x*_*n*1_(*t* = 1) was sampled from a uniform distribution U(0.1, 0.5). The abundance of species *i* in individual *n* at time *t* was calculated as follows:

$$x_{\mathrm{ni}}\left(t+∆t\right)=\eta_{\mathrm{ni}}\left(t\right)x_{\mathrm{ni}}\left(t\right)\text{exp}\;\left(∆t\sum_{j}c_{\mathrm{nij}}\left(x_{\mathrm{nj}}\left(t\right)-\langle x_{\mathrm{nj}}\rangle \right)\right).$$

We simulated one subject (*n* = 1) over 50 time points (*t* ∈ {1, 2, …, 50}). A total of *P* species (*i,j* = 1, *…, P*) were included in the simulations. The equilibrium abundance $\langle x_{1}\rangle$ was fixed at 0.3, and the sampling interval was set to ∆t = 1. To simplify calculations, stochastics effects $\eta_{\mathrm{nt}}$(*t*) were ignored. Subsequently, we evaluated the performance of *MicrobTiSDA* by comparing the inferred interspecies interaction coefficients from the test data set with the known ground‐truth coefficients.

We designed 15 parameter configurations (varying the number of species P, and interaction matrix sparsity dens) (Table S1) to systematically evaluate *MicrobTiSDA* under different simulation conditions. The results showed that *MicrobTiSDA* exhibits strong discriminative ability in identifying the presence of interspecies interactions (mean area under the receiver operating characteristic curve = 0.742; Figure 2A). Moreover, the inferred interaction coefficients were quantitatively close to the ground truth (mean MAE = 0.249, mean MSE = 0.152; Figure 2B). However, the overall performance in predicting the direction (sign) of interactions was limited (mean SignAcc = 0.427; Figure 2B), indicating that, on average, only 42.7% of interspecies interaction signs were correctly inferred. Further analysis revealed that sign prediction accuracy improved markedly under sparse interaction matrix (dens = 0.01), with SignAcc > 0.6 (Table S1). Conversely, inference performance declined with increasing species number. Together, these findings indicate that *MicrobTiSDA* provides reliable inference of microbial interactions, particularly in communities with relatively low species richness.

**Figure 2:**
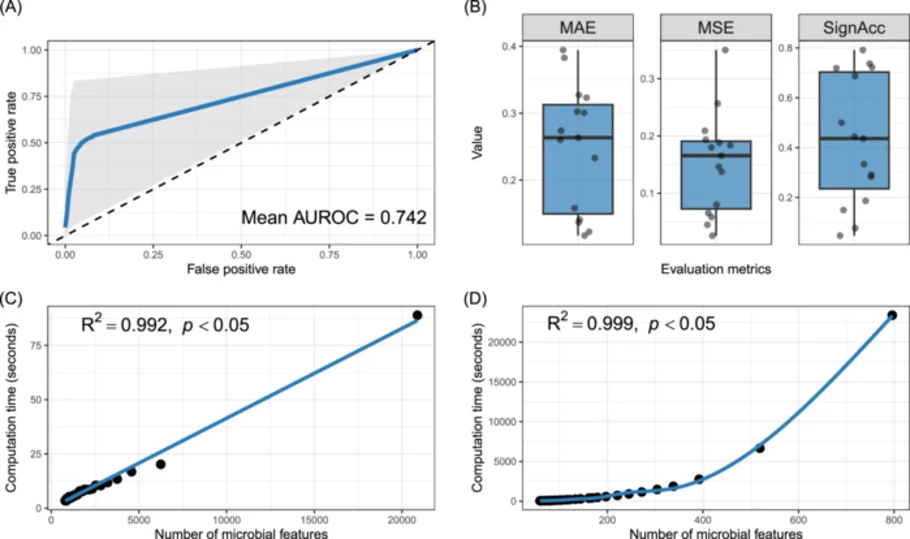
Performance evaluation of *MicrobTiSDA*. (A) Mean receiver operating characteristics curve for interspecies interaction inference based on simulated data. (B) Boxplots summarizing mean absolute error (MAE), mean squared error (MSE), and sign accuracy (SignAcc) for interspecies interaction inference. (C) Runtime of regression model fitting module across simulated datasets with increasing number of microbial features (796 to 20,884 microbial features). (D) Runtime of the interspecies interaction inference module across simulated datasets with different feature numbers (59 to 796 microbial features).

Furthermore, to assess the robustness of *MicrobTiSDA* in inferring species interactions under the discrete‐time Lotka–Volterra framework, we performed a parameter sensitivity analysis. Specifically, we inferred interaction networks under varying parameter settings (abundance centering methods: mean and median; MSE thresholds ranging from 10^−1^ to 10^−6^) and calculated Spearman correlations among the resulting interaction matrices. The results showed that interaction patterns remained highly consistent across parameter combinations, with the lowest Spearman correlation coefficient exceeding 0.7 (Figure S1). These findings indicate that the inferred interactions are not sensitive to parameter selection, thereby supporting the robustness of results derived from the linear model assumption.

## 4 EVALUATION OF COMPUTATIONAL EFFICIENCY

To assess the computational efficiency of *MicrobTiSDA* under high‐dimensional settings, we used a longitudinal infant gut microbiome data set from de Muinck et al. [12], which monitored infants during their first year of life. We selected samples from a pair of dizygotic twins collected between days 6 and 60 postpartum. The original data set included 77 stool samples (34 from individual ID10, 43 from ID11) and 476,921 microbial features. After interpolation, we reconstructed continuous time‐series for both individuals, each comprising 55 time points. All analyses were performed on a MacBook Pro (Apple M1 chip, 8‐core CPU, 16 GB RAM).

Given that the core functionalities of *MicrobTiSDA* include inferring interspecies interactions and natural spline regression modeling, we focused on evaluating the computational performance of these two modules under high‐dimensional conditions. For regression modeling task, we applied the minimum total abundance filtering thresholds (0 to 100, in steps of 2) to generate 50 test subsets, each containing 110 samples and varying numbers of microbial features (796 to 20,884). A highly significant linear relationship was observed between runtime and the number of features (*R* ^2^ = 0.992, *p* < 0.05; Figure 2C), indicating scalable computational cost for high‐dimensional regression tasks.

For interaction inference, the bagging strategy used to enhance robustness incurs higher computational costs. We therefore applied more stringent filtering (total abundance thresholds from 1000 to 3000, step size 100), yielding 30 test subsets with 110 samples and 59 to 796 microbial features. Runtime increase nonlinearly with feature numbers (*R* ^2^ = 0.999, *p* < 0.05; Figure 2D), reflecting the growing computational demand in higher dimensions. These results underscore that with appropriate feature filtering, *MicrobTiSDA* remains computationally feasible for interaction inference.

In summary, our evaluation demonstrates that *MicrobTiSDA* achieves high computational scalability in regression modeling, while interspecies interaction inference requires a balance between feature dimensionality and available computational resources. These performance characteristics support the practical utility of *MicrobTiSDA* for large‐scale microbiome time‐series analysis.

## 5 COMPREHENSIVE APPLICATION OF *MICROBTISDA*

Following systematic evaluation of *MicrobTiSDA*'s performance and computational efficiency, we applied the tool to three real‐world microbiome datasets (detailed in Supporting Information): an in vitro aquatic microbiome, infant gut microbiomes, and a preterm infant gut microbiome data set associated with sepsis. In the aquatic microbiome, *MicrobTiSDA* revealed temporal abundance dynamics across eight independent replicate experiments (Figures S2−S10), inferred interspecies interactions, and identified keystone taxa that potentially regulate community dynamics (Figure S11). For a pair of dizygotic twins, we characterized gut microbiota temporal dynamics from day 6 to day 60 after birth and pinpointed individual‐specific keystone species (Figures S12−S14). Finally, using the random forest classification module, we identified microbial biomarkers that effectively discriminate preterm infants with pathogenic *Escherichia coli*‐associated sepsis from matched controls and compared their temporal abundance patterns between the cohorts (Figures S15 and S16). These analyses demonstrate that *MicrobTiSDA* reliably infers interspecies interactions and accurately captures time‐specific abundance dynamics across diverse microbiome time‐series datasets, highlighting its flexibility and applicability in both ecological and clinical contexts.

Longitudinal microbiome analysis is essential for elucidating the temporal dynamics of host‐associated and environmental microbial communities [13, 14]. However, data processing and analysis—particularly for inferring interspecies interactions and characterizing temporal patterns of microbial feature abundance—remain challenges, especially for researchers with limited bioinformatics expertise [15, 16]. The complexity is further compounded by the need to integrate outputs from different tools and processing steps. To address these challenges and streamline longitudinal microbiome analysis, we developed *MicrobTiSDA*, a highly integrated and workflow‐oriented R package that encompasses the entire analytical pipeline, from data preprocessing and transformation to statistical modeling and visualization. By streamlining the entire analytical pipeline, *MicrobTiSDA* enables reproducible, user‐friendly, and comprehensive longitudinal microbiome analyses. The source code and detailed usage example of *MicrobTiSDA* are publicly available on GitHub (https://github.com/Lishijiagg/MicrobTiSDA).

## AUTHOR CONTRIBUTIONS

**Shijia Li**: Writing—original draft; writing—review and editing; conceptualization; formal analysis; validation; methodology; software; data curation; visualization. **Remco Kort**: Writing—review and editing; supervision. **Tim G. J. de Meij**: Writing—review and editing; data curation. **Stanley Brul**: Writing—review and editing; project administration; supervision. **Meike T. Wortel**: Writing—review and editing; conceptualization; supervision; **Johan A. Westerhuis**: Writing—original draft; writing—review and editing; conceptualization; methodology; supervision. All authors have read the final manuscript and approved it for publication.

## CONFLICT OF INTEREST STATEMENT

The authors declare no conflicts of interest.

## ETHICS STATEMENT

No information regarding participants' socioeconomic status, ethnicity, or ancestry was collected. The collection of samples and data from participants was conducted in accordance with the guidelines of the Ethics Committee of the Amsterdam UMC (2014.386).

## Supporting information


**Figure S1.** Heatmap of Spearman correlation coefficients showing the consistency of inferred interspecies interaction matrices across 12 parameter configurations.
**Figure S2.** The hierarchical clustering results of the OTU temporal patterns for 8 replicate experimental in vitro microbiomes.
**Figure S3.** Visualizations of clusters of ASVs with similar temporal trends in the Rep.1 cultivation of the in vitro aquatic microbiome.
**Figure S4.** Visualizations of clusters of ASVs with similar temporal trends in the Rep.2 cultivation of the in vitro aquatic microbiome.
**Figure S5.** Visualizations of clusters of ASVs with similar temporal trends in the Rep.3 cultivation of the in vitro aquatic microbiome.
**Figure S6.** Visualizations of clusters of ASVs with similar temporal trends in the Rep.4 cultivation of the in vitro aquatic microbiome.
**Figure S7.** Visualizations of clusters of ASVs with similar temporal trends in the Rep.5 cultivation of the in vitro aquatic microbiome.
**Figure S8.** Visualizations of clusters of ASVs with similar temporal trends in the Rep.6 cultivation of the in vitro aquatic microbiome.
**Figure S9.** Visualizations of clusters of ASVs with similar temporal trends in the Rep.7 cultivation of the in vitro aquatic microbiome.
**Figure S10.** Visualizations of clusters of ASVs with similar temporal trends in the Rep.8 cultivation of the in vitro aquatic microbiome.
**Figure S11.** Interaction topologies of aquatic microbiomes of eight replicate experiments. Rep.1 to Rep.8 represent replicate experiments 1‐8.
**Figure S12.** Results of infant gut microbiome dataset analysis via *MicrobTiSDA*.
**Figure S13.** Visualizations of clusters of OTUs with similar temporal trends in the ID10's gut microbiota of the infant gut microbiome dataset.
**Figure S14.** Visualizations of clusters of OTUs with similar temporal trends in the ID11's gut microbiota of the infant gut microbiome dataset.
**Figure S15.** Random Forest classification results and temporal pattern clustering of microbial features in the preterm infant sepsis gut microbiome dataset.
**Figure S16.** Visualizations of clusters of OTUs with similar temporal trends in the control and sepsis group of the Gut microbiome dataset of preterm infant with sepsis.
**Figure S17.** Schematic figure of the Learning Interactions from MIcrobial Time Series (LIMITS) algorithm.
**Figure S18.** Sample information for the dataset of 4 septic preterm infants (ID_7, ID_14, ID_19, ID_21) and 4 individually matched control preterm infants (ID_31, ID_37, ID_42, ID_43).

**Table S1.** Simulation configurations and performance of MicrobTiSDA in inferring interspecies interactions.
**Table S2.** Parameter configurations used in the sensitivity analysis of MicrobTiSDA.
**Table S3.** Prevalence of the selected top 10 biomarker OTUs in sepsis and control groups.

## Data Availability

The datasets generated and analyzed during the current study are available on GitHub at: https://github.com/Lishijiagg/MicrobTiSDA. The data and scripts used are saved in GitHub https://github.com/Lishijiagg/MicrobTiSDA/tree/main/data. Supplementary materials (methods, figures, tables, graphical abstract, slides, videos, Chinese translated version, and update materials) may be found in the online DOI or iMeta Science http://www.imeta.science/imetaomics/.
