# Supplementary material for: MicrobTiSDA: A flexible R package for inferring interspecies interactions and abundance dynamics in microbiome time‐series data
Source: IMetaOmics. 2025 Nov 27;3(1):e70067. doi: 10.1002/imo2.70067 (PMC13674398; doi:10.1002/imo2.70067)
Supplement: Supplementary file 1 [file IMO2-3-e70067-s001.docx]

Supporting information to

**MicrobTiSDA: a flexible R package for inferring interspecies interactions and abundance dynamics in microbiome time-series data**

**Running title**: MicrobTiSDA: an R package for microbiome time-series data analysis

Shijia Li^1, 2^, Remco Kort^3^, Tim G.J. de Meij^4^, Stanley Brul^1^, Meike T. Wortel^1*^, Johan A. Westerhuis^2*^

^1^Molecular Biology & Microbial Food Safety, Swammerdam Institute for Life Science, University of Amsterdam, Amsterdam, 1090 GE, The Netherlands

^2^Biosystem Data Analysis, Swammerdam Institute for Life Science, University of Amsterdam, Amsterdam, 1090 GE, The Netherlands

^3^Amsterdam Institute for Life and Environment (A-LIFE), Vrije Universiteit Amsterdam, Amsterdam, 1081 HV, The Netherlands

^4^Amsterdam Gastroenterology Endocrinology Metabolism Research Institute, Amsterdam UMC, Amsterdam, 1105 AZ, The Netherlands

^*^Correspondence: m.t.wortel@uva.nl (Meike T. Wortel), [j.a.westerhuis@uva.nl](mailto:j.a.westerhuis@uva.nl) (Johan A. Westerhuis)

**METHODS**

**Data preprocessing**

Data Filtering

*MicrobTiSDA* begins by filtering the input high-throughput sequencing data (OTU/ASV count table). In a high-dimensional count table, low-abundant and low-prevalent features often exhibit irregular or noisy patterns over time, making them unsuitable for time-series analysis based on regression models. To address this, *MicrobTiSDA* provides a flexible microbial feature filtering approach, enabling users to define two key thresholds based on the scale of the input dataset: the minimum total abundance of microbial features across all samples, and the minimum prevalence of microbial features within individual subjects across all time-series samples. This dual-threshold filtering strategy, which is tailored to the scale of the input dataset, effectively helps users filter out noisy microbial features.

Data Interpolation

Missing time points are a common limitation in microbiome time-series studies [1]. These missing points result in inconsistent sample numbers across subjects and variations in sampling times, thereby complicating data analysis. To address this issue, *MicrobTiSDA* employs Piecewise Cubic Hermite Interpolation (*PCHIP*) [2], aiming to generate a more complete and uniform data for downstream analysis. This method fits cubic polynomials between adjacent time points in each subject time-series data while ensuring the continuity of both the function and its derivative, enabling interpolation for missing time points and generating continuous time-series data for individual subjects.

Data Transformation

Differences in sequencing depth across samples can result in abundance data that are not directly comparable. Therefore, data transformation is required. *MicrobTiSDA* utilizes the Modified Centered Log Ratio (MCLR) transformation method [3]. The MCLR method calculates the geometric mean of each sample from positive proportions only, then normalizes and log-transforms all non-zero proportions by using that geometric mean and applies an identical shift operation to all non-zero components in the dataset. Specifically, let $x_{nt}\in\Omega^{I}$ denote the compositional vector for the sample from subject $n (n = 1:N)$ at timepoint $t (t = 1:T)$, where $\Omega^{I}$ represents the collection of $I(i=1:I)$ microbial features. For simplicity of illustration, assume that the first $q$ elements of $x_{nt}$ are zero while the remaining elements are non-zero. Then, it can be expressed as:

$$\begin{aligned} {mclr}_{\varepsilon}\left( x_{nt} \right)=[0,\ldots,0,\ln\frac{x_{nt(q+1)}}{\tilde{g}\left( x_{nt} \right)}+\varepsilon,\ldots,\ln\frac{x_{ntI}}{\tilde{g}\left( x_{nt} \right)}+\varepsilon]\#\left( 1 \right) \end{aligned}$$

where $\tilde{g}\left( x_{nt} \right)={(\prod_{i=q+1}^{I} x_{nti})}^{1/{(I-q)}}$ is the geometric mean of the non-zero elements of $x_{nt}$. $\varepsilon$ is a positive value, here define $z_{min}=\min\left\{ \ln\frac{x_{nt(q+1)}}{\tilde{g}(x_{nt})},\ldots,\ln\frac{x_{I}}{\tilde{g}(x_{nt})} \right\}$, the constraint $\varepsilon>\left| z_{min} \right|$ ensures that $z_{nt(q+1)},\ldots, z_{ntI}$ are strictly positive. The default choice of $\varepsilon=\left| z_{min} \right|+c$, with $c=0.1$. The MCLR transformation is invariant to the addition of extra zero elements, preserves the original zero measurements, and is overall rank preserving.

**Interspecies interaction inference**

To describe interspecies dynamics, *MicrobTiSDA* adopts the discrete-time Lotka-Volterra (dLV) model [4], linking the abundance of species $i$to subject $n$ at time $t+\Delta t$, with the abundance of all species for that subject $n$ at time $t$, $X_{n}=\left\{ x_{n1}\left( t \right),x_{n2}\left( t \right),\ldots,x_{nI}(t) \right\}$. The dLV model is mathematically represented as:

$$\begin{aligned} x_{ni}\left( t+\Delta t \right)=\eta_{ni}\left( t \right)x_{ni}\left( t \right)\exp\left( \Delta t\sum_{j} c_{nij}\left( x_{nj}\left( t \right)-\left\langle x_{nj} \right\rangle\right) \right)\#\left( 2 \right) \end{aligned}$$

where $\left\langle x_{nj} \right\rangle$ is the equilibrium abundance of species $j (j\in I)$, typically defined as the median abundance across all samples of subject $n$; $c_{nij}$ represents the interaction coefficient of species $j$ on species $i$ for subject $n$ ; and $\eta_{ni}\left( t \right)$ accounts for a log-normally distributed multiplicative noise, which indicates environmental and demographic stochastic effects on species $i$ in the microbiome of the subject.

In this study, to simplify calculations, we ignore stochastics effects $\eta_{nt}(t)$, set $\Delta t=1$, and use MCLR transformed data. Taking the natural logarithm of equation 2 yields:

$$\begin{aligned} \ln x_{ni}\left( t+1 \right)-\ln x_{ni}\left( t \right)=\sum_{j} c_{nij}\left( x_{nj}\left( t \right)-\left\langle x_{nj} \right\rangle\right)\#\left( 3 \right) \end{aligned}$$

The logarithmic changes in abundances and the deviations from the equilibrium abundances in equation 3 can be expressed in matrix form:

$$\begin{aligned} V_{n}=D_{n}\cdot C_{n}\#\left( 4 \right) \end{aligned}$$

where:

- $V_{n}$ is the matrix of species logarithmic abundance changes, with dimensions $\left( T-1 \right)\times I$. Here, $T$ is length of the time series, and $I$ is the number of species. Its elements are calculated as:

$$\begin{aligned} V_{n}=\left[ \begin{matrix} \ln x_{n1}\left( 2 \right)-\ln x_{n1}\left( 1 \right) & \cdots& \ln x_{nI}\left( 2 \right)-\ln x_{nI}\left( 1 \right) \\ \vdots& \ddots& \vdots\\ \ln x_{n1}\left( T \right)-\ln x_{n1}\left( T-1 \right) & \cdots& \ln x_{nI}\left( T \right)-\ln x_{nI}\left( T-1 \right) \end{matrix} \right]\#\left( 5 \right) \end{aligned}$$

- $D_{n}$ is the design matrix of species abundances deviating from their equilibrium values, with dimensions $(T-1)\times I$:

$$\begin{aligned} D_{n}=\left[ \begin{matrix} x_{n1}\left( 1 \right)-\left\langle x_{n1} \right\rangle& \cdots& x_{nI}\left( 1 \right)-\left\langle x_{nI} \right\rangle\\ \vdots& \ddots& \vdots\\ x_{n1}\left( T-1 \right)-\left\langle x_{n1} \right\rangle& \cdots& x_{nI}\left( T-1 \right)-\left\langle x_{nI} \right\rangle\end{matrix} \right]\#\left( 6 \right) \end{aligned}$$

- $C_{n}$ is the interaction coefficient matrix, with dimensions $I\times I$:

$$\begin{aligned} C_{n}=\left[ \begin{matrix} c_{n11} & \cdots& c_{n1I} \\ \vdots& \ddots& \vdots\\ c_{nI1} & \cdots& c_{nII} \end{matrix} \right]\#\left( 7 \right) \end{aligned}$$

The regression coefficient matrix $C_{n}$ is estimated using the pseudo-inverse matrix:

$$\begin{aligned} C_{n}=D_{n}^{+}V_{n}\#\left( 8 \right) \end{aligned}$$

where the $+$ denotes the pseudo-inverse.

To enhance the sparsity and interpretability of the results, *MicrobTiSDA* incorporates the LIMITS algorithm (Figure S17), which identifies species interactions through stepwise regression, producing a sparse interaction coefficient matrix $C_{n}$.

The steps of the LIMITS algorithm are as follows:

1. Random splitting of training and testing datasets:

In each iteration, 50% of the samples of each subject are randomly selected as the training set, and the remaining 50% are used as the test set (Figure S17A).

2. Initial regression model:

For each species $i$ in subject $n$, construct a linear regression including only the self-interaction term $c_{nii}$:

$$\begin{aligned} \ln x_{ni}\left( t+1 \right)-\ln x_{ni}\left( t \right)=c_{nii}\left( x_{ni}\left( t \right)-\left\langle x_{ni} \right\rangle\right)\#\left( 9 \right) \end{aligned}$$

Then calculate the mean squared error (MSE) on the test set:

$$\begin{aligned} MSE=\frac{1}{T_{test}}\sum_{t\in T_{test}} \left[ c_{nii}\left( x_{ni}\left( t \right)-\left\langle x_{ni} \right\rangle\right)-\left( \ln x_{ni}\left( t+1 \right)-\ln x_{ni}\left( t \right) \right) \right]^{2}\#\left( 10 \right) \end{aligned}$$

3. Stepwise regression to add interaction terms:

Define the remaining species as the candidate variable set $S$. Using stepwise regression, iteratively add candidate species $j\neq i$ to the model, forming a new regression model:

$$\begin{aligned} \ln x_{ni}\left( t+1 \right)-\ln x_{ni}\left( t \right)=c_{nii}\left( x_{ni}\left( t \right)-\left\langle x_{ni} \right\rangle\right)+\sum_{j\in S} c_{nij}\left( x_{nj}\left( t \right)-\left\langle x_{nj} \right\rangle\right)\#\left( 11 \right) \end{aligned}$$

Predict the outcome on the test set, compute the new mean square error (MSE), and evaluate the improvement rate:

$$\begin{aligned} \theta=\frac{{MSE}_{before}-{MSE}_{after}}{{MSE}_{before}}\#\left( 12 \right) \end{aligned}$$

$\theta$ refers to how much the MSE evaluated on the test dataset decreases. If $\theta$ is above the predefined improvement threshold (default 10^-3^), include the candidate species $j$ in the current model (Figure S17B).

4. Bagging to enhance robustness:

Repeat the above step $B$ times (Bagging iterations), randomly splitting the training and testing sets in each iteration and performing stepwise regression (Figure S17C).

5. Final interaction coefficient matrix:

Compute the median of the interaction coefficients $c_{nij}^{(B)}$ across all interactions to generate the final sparse interaction coefficient matrix:

$$\begin{aligned} c_{nij}=median\left( c_{nij}^{\left( 1 \right)},c_{nij}^{\left( 2 \right)},\ldots,c_{nij}^{\left( B \right)} \right)\#\left( 13 \right) \end{aligned}$$

Note: Interaction coefficients with $\left| c_{nij} \right|<0.0001$ were ignored.

6. Identification of keystone species:

Beyond reconstructing the sparse interaction network, *MicrobTiSDA* also enables the identification of potential keystone species. Specifically, user can manually define a minimum interaction degree threshold, corresponding to the minimum number of non-zero interaction coefficients a species must maintain with other species. Species that satisfy this criterion are considered keystone species, as their abundance dynamics are inferred to influence a broader set of microbial features within the microbial community.

To systematically evaluate the performance of *MicrobTiSDA* in inferring interspecies interactions from temporal microbiome data, we established a benchmarking pipeline based on the dLV model. Specifically, given the number of species $p$ and s predefined interaction network sparsity $dens$, we first generated a ground-truth interaction coefficient matrix $C_{n,true}\in\Omega^{p\times p}$ with a proportion of nonzero coefficients determined by $dens$ and negative diagonal elements to represent self-inhibition. Subsequently, abundance trajectories of length $T$, ${\{x_{n}(t)\}}_{t=1}^{T}$ (each $x_{n}(t)\in\Omega^{T}$), were simulated according to Eq. 2 and used as observed data. We then applied the LIMITS algorithm to the simulated datasets to infer interaction coefficients and obtained an estimated matrix $C_{n}$. The inferred $C_{n}$ was compared against the ground truth $C_{n,true}$ to assess the inference accuracy of MicrobTiSDA.

The evaluation covered three aspects:

1. Structural recovery:

The edge strength score $c_{nij}$ between species $x_{ni}(t)$ and $x_{nj}(t)$ was used to construct ROC curves

$$\begin{aligned} TPR=\frac{TP}{TP+FN}, FPR=\frac{FP}{FP+TN}, AUROC=\int_{0}^{1} TPRd\left( FDR \right) \#\left( 14 \right) \end{aligned}$$

2. Parameter estimation accuracy:

For the set of true edges $E_{n}=\left\{ [x_{ni}\left( t \right),x_{nj}(t)]:c_{nij,true}\neq0 \right\}$, we quantified the mean squared error (MSE) and mean absolute error (MAE)

$$\begin{aligned} {MSE}_{nij}=\frac{1}{\left| E_{n} \right|}\sum_{[x_{ni}\left( t \right),x_{nj}(t)]\in E_{n}} \left( c_{nij}-c_{nij,true} \right)^{2}\#\left( 15 \right) \end{aligned}$$

$$\begin{aligned} {MAE}_{nij}=\frac{1}{\left| E_{n} \right|}\sum_{[x_{ni}\left( t \right),x_{nj}(t)]\in E_{n}} \left| c_{nij}-c_{nij,true} \right|\#\left( 16 \right) \end{aligned}$$

3. Interaction sign consistency:

To evaluate whether the predicted interaction directions matched the true signs, we defined

$$\begin{aligned} {SignAcc}_{nij}=\frac{1}{\left| E_{n} \right|}\sum_{[x_{ni}\left( t \right),x_{nj}(t)]\in E_{n}} 1\left[ sign\left( c_{nij} \right)=sign\left( c_{nij,true} \right) \right]\#\left( 17 \right) \end{aligned}$$

**Regression model fitting**

*MicrobTiSDA* can be employed to analyse the relationship between microbiota abundances and time in two distinct scenarios: A) time-series data of the microbiome from a single subject, B) time-series data from groups that encompass multiple subjects.

For individual-level studies, *MicrobTiSDA* incorporates natural spline regression to construct smooth terms within the Generalized Additive Model (GAM) [5] , enabling flexible modeling of non-linear temporal patterns in microbial abundances. In this approach, the natural spline functions serve as smooth basis functions within the GAM, allowing for flexible and interpretable modeling of microbial abundance trends over time. The model is formulated as:

$$\begin{aligned} x_{ni}\left( t \right)= \beta_{n0}+\sum_{k=1}^{K} \beta_{nk}\cdot N_{nk}\left( t \right)+\epsilon_{ni}\left( t \right)\#\left( 18 \right) \end{aligned}$$

where $x_{ni}(t)$ represents the abundance of microbial feature $i$ for subject $n$ at time point $t$, $\beta_{n0}$ is the intercept term, $\beta_{nk}$ denotes the regression coefficients for the $k$-th natural spline basis function $N_{nk}(t)$, and $\epsilon_{ni}$ is the error term. The parameter $K$ refers to the number of basis functions, which is equal to the number of knots plus one (*i.e.* $K=number of knots+1$).

For group-level studies, *MicrobTiSDA* utilizes a Mixed-Effect Natural Spline Regression Model to fit the overall temporal dynamics of microbial features within each group. Assuming the dataset contains $m$ groups, each with $n$ subjects, the model is formulated as:

$$\begin{aligned} x_{n(m)i}\left( t \right)=\beta_{m0}+\sum_{k=1}^{K} \beta_{mk}\cdot N_{mk}(t)+b_{n\left( m \right)}+\epsilon_{n(m)i}(t)\#\left( 19 \right) \end{aligned}$$

where $x_{n(m)i}\left( t \right)$ represents the abundance of microbial feature $i$ at time point $t$ in group $m$. The random effect $b_{n\left( m \right)}$ reflects the departure of individual $n$ in group $m$ from overall population average effects.

In both scenarios, users can specify knot locations based on prior knowledge, such as treatment or environmental exposure time points. If no prior information is provided, the optimal number and positions of the knots are determined using the Generalized Cross-Validation (GCV) criterion, ensuring a data-driven approach to modeling temporal dynamics.

To evaluate the time-series fitting performance of the natural spline regression implemented in *MicrobTiSDA*, we employed a 10-fold cross-validation framework and compared it with the three commonly used regression approaches (1) stepwise polynomial regression (*maSigPro*), (2) smoothing spline regression (*metaDprof*), and (3) locally weighted regression (*LOWESS*). Model performance was assessed using five commonly applied metrics:

1. Root Mean Squared Error (RMSE)

$$\begin{aligned} {RMSE}_{ni}=\sqrt{\frac{1}{T}\sum_{t=1}^{T} \left( x_{nit}-\hat{x}_{nit} \right)^{2}}\#\left( 20 \right) \end{aligned}$$

2. Mean Absolute Error (MAE)

$$\begin{aligned} {MAE}_{ni}=\frac{1}{T}\sum_{t=1}^{T} \left| x_{nit}-\hat{x}_{nit} \right|\#\left( 21 \right) \end{aligned}$$

3. Mean Absolute Percentage Error (MAPE)

$$\begin{aligned} {MAPE}_{ni}=\frac{100}{T}\sum_{t=1}^{T} \left| \frac{x_{nit}-\hat{x}_{nit}}{x_{nit}} \right|\#\left( 22 \right) \end{aligned}$$

4. Coefficient of Determination (R^2^)

$$\begin{aligned} R_{ni}^{2}=1-\frac{\sum_{t=1}^{T} \left( x_{nit}-\hat{x}_{ni} \right)^{2}}{\sum_{t=1}^{T} \left( x_{nit}-\bar{x}_{ni} \right)^{2}}\#\left( 23 \right) \end{aligned}$$

5. Prediction Bias (Bias)

$$\begin{aligned} {Bias}_{ni}=\frac{1}{T}\sum_{t=1}^{T} \left( \hat{x}_{nit}-x_{nit} \right)\#\left( 24 \right) \end{aligned}$$

Where $x_{nit}$ denotes the abundance of microbial feature $i$ for subject $n$ at time point $t$, $\hat{x}_{nit}$ the predict value, $\bar{x}_{ni}$ the mean of abundances. For each fold, the training set was used for model fitting, while the test set was used for independent performance assessment.

**Temporal patterns clustering**

After constructing the regression models, *MicrobTiSDA* fits species temporal trends based on the predicted abundance values of each regression model to describe the dynamic trends of microbial features. To evaluate the similarity between temporal patterns of microbial features within the same group or subject, *MicrobTiSDA* calculates the correlation distance for the individual-level using the formula:

$$\begin{aligned} d_{corr}n\left( i,j \right)=1-\frac{\sum_{t=1}^{T} \left( x_{nit}-\bar{x}_{ni} \right)\left( x_{njt}-\bar{x}_{nj} \right)}{\sqrt{\sum_{t=1}^{T} \left( x_{nit}-\bar{x}_{ni} \right)^{2}}\sqrt{\sum_{t=1}^{T} \left( x_{njt}-\bar{x}_{nj} \right)^{2}}}\#\left( 25 \right) \end{aligned}$$

And for the group-level:

$$\begin{aligned} d_{corr}m\left( i,j \right)=1-\frac{\sum_{n\in m} \sum_{t=1}^{T} \left( x_{nit}-\bar{x}_{ni} \right)\left( x_{njt}-\bar{x}_{nj} \right)}{\sqrt{\sum_{n\in m} \sum_{t=1}^{T} \left( x_{nit}-\bar{x}_{ni} \right)^{2}}\sqrt{\sum_{n\in m} \sum_{t=1}^{T} \left( x_{njt}-\bar{x}_{nj} \right)^{2}}}\#\left( 26 \right) \end{aligned}$$

Using the correlation distance, *MicrobTiSDA* employs hierarchical clustering to group microbial features and generates a clustering dendrogram. Users can manually specify a threshold (default: 0.15) for the correlation distance to prune the dendrogram and determine the optimal number of clusters. If no threshold is specified, *MicrobTiSDA* automatically selects the optimal number of clusters by calculating the highest silhouette coefficient [6]. The resulting clusters reveal microbial features with similar dynamic patterns. Furthermore, *MicrobTiSDA* can identify groups of microbial features that are negatively correlated with a given microbial feature based on correlation distance. This allows the inference of microbial feature groups with opposite dynamic patterns.

**Microbial features-based random forest classification**

When analysing time-series data across different groups, *MicrobTiSDA* employs a random forest classification method to identify key features associated with specific groups (*e.g.*, treatment or disease stages) [7]. This approach reduces dimensionality while retaining the most informative microbial features, which are more likely to exhibit certain temporal patterns. First, *MicrobTiSDA* preprocesses the data by removing low-abundant features with limited contributions to classification, thereby improving computational efficiency. The filtered data is then split into a training set (default 70% samples) and a test set (default 30% samples). The random forest algorithm is applied to the training set to build the classification model, which is subsequently evaluated on the test set. The microbial features that contributed most to the performance of the classification model were screened by 5 repetitions of 10-fold cross-validation. Users can select the optimal number of important features based on their rankings by Mean Decrease Accuracy (MDA). The selected key features can be visualized using Non-Metric Multidimensional Scaling (NMDS) to illustrate the classification results and sample group distributions.

**Visualization**

MicrobTiSDA incorporates a comprehensive visualization suite built upon *ggplot2* and visNetwork, designed to facilitate both the presentation and interpretation of analytical results. The visualization functionalities include:

1. Graphical outputs of random forest model training and prediction, enabling an intuitive evaluation of classification performance.
2. Interactive network representations of inferred microbial interactions, allowing exploration of community topology and key species.
3. Dendrograms of microbial feature time-series clustering results, highlighting similarity patterns and structural relationships.
4. Trend curve visualizations of microbial features exhibiting similar (or opposing) temporal dynamics, supporting in-depth characterization of species abundance trajectories.

Together, these visualization capabilities provide users with a powerful and interpretable framework for analyzing longitudinal microbiome data, thereby enhancing both the accessibility and explanatory depth of the results.

**Example datasets**

The in vitro aquatic microbiome dataset

The first case study utilized the in vitro microbiome dataset published by Fujita *et* *al.* [8]. This dataset comprises 48 experimental microbiomes derived from 6 different experimental conditions: two sources of microbiomes (soil and aquatic) and three types of culture media (oatmeal, oatmeal-peptone, and peptone). Each condition includes eight replicates, with each replicate continuously monitored for 110 days to observe the dynamics of the microbiomes. For this case study, we focused on a single experimental condition – aquatic microbiomes cultured in peptone medium. This subset of the data includes a total of 880 samples and 29 Amplicon Sequence Variants (ASVs), with each condition contributing between 5 and 11 ASVs.

The infant gut microbiome dataset

The second case study investigates the colonization process of the infant gut microbiome [9]. This research analyzed 2684 fecal samples from 12 infants during the first year of life, encompassing 376,921 Operational Taxonomic Units (OTUs), thereby providing insights into the human gut colonization process. In this case study, we focus on the dynamic changes in the gut microbiome of a pair of dizygotic twins (ID10 and ID11) from day 6 to day 60 after birth. Fecal samples from ID10 and ID11 were collected starting on day 6 post-birth, with a total of 34 and 43 samples collected, respectively, by day 60. Additionally, ID10 was hospitalized due to *Streptococcus* serogroup B $\beta$-hemolytic *(S. agalactiae*) and received antibiotic treatment from day 22 to day 33.

The preterm infant gut microbiome dataset associated with sepsis

The third case study involved four preterm infants with culture-confirmed *E. coli* sepsis (ID_7, ID_14, ID_19, ID_21) and four matched preterm controls without sepsis (ID_31, ID_37, ID_42, ID_43), all enrolled from a preterm birth cohort established at Amsterdam UMC. The study was approved by the institutional ethics committee (2014.386), and written informed consent was obtained from the parents/legal caretakers. Fecal samples and clinical data were collected daily from birth until day 28, and samples were stored immediately after collection at –20°C. Matching was performed based on gestational age, feeding pattern, antibiotic exposure, and postnatal age. The day on which the diagnostic work-up, including blood culture, confirmed *E. coli* sepsis was defined as the onset day of sepsis (*t* = 0). To standardize temporal profiles across individuals, the sepsis diagnosis day was aligned to pseudo-Day 11, with sampling days adjusted accordingly to a 10-day window prior to diagnosis (pseudo-Days 1–10). Each infant contributed between 5 and 9 fecal samples, resulting in a total of 51 samples (Figure S18).

DNA was extracted using the QIAamp PowerFecal DNA Kit (Qiagen, Hilden, Germany). Sample disruption was performed using the TissueLyser II (Qiagen, Hilden, Germany) for 2 min at 30 Hz. DNA was eluted in 50 μl of elution buffer. Negative extraction controls were included in each batch to monitor for contamination. Extracted DNA was stored at 2–8°C until quantification and downstream applications. DNA concentration was measured using the Qubit™ 3 Fluorometer (Invitrogen, Thermo Fisher Scientific, Waltham, MA, USA) with the dsDNA Broad Range (BR) Assay Kit. DNA samples were normalized to a concentration of 5 ng/μl prior to PCR.

Amplification and sequencing of the 16S rRNA gene were conducted by the Department of Cancer and Genomic Sciences at the University of Birmingham, following the Earth Microbiome Project protocol [10,11]. The V4 hypervariable region of the 16S rRNA gene was amplified using a custom-designed 515F forward primer and 806R reverse primer (both from Sigma-Aldrich, Saint Louis, MO, USA) in a one-step, single-indexed PCR approach.

Each 50 μl PCR reaction consisted of 47 μl master mix containing 34.6 μl nuclease-free water (Qiagen, Hilden, Germany), 10 μl 5× Platinum™ II PCR Buffer, 1 μl dNTP mix, 1 μl reverse primer (10 μM final concentration), 0.4 μl Platinum™ II Taq Hot-Start DNA Polymerase (all Thermo Fisher Scientific, Waltham, MA, USA), 2 μl template DNA (5 ng/μl), and 1 μl forward primer (10 μM final concentration).

PCR conditions were as follows: initial denaturation at 94°C for 3 min, followed by 35 cycles of 94°C for 45 s, 50°C for 60 s, and 72°C for 90 s, with a final extension at 72°C for 10 min and a hold at 4°C. Appropriate negative and positive controls were included throughout both DNA extraction and amplification procedures.

Post-PCR cleanup was performed using AMPure XP magnetic beads (A63882, Beckman Coulter, Brea, CA, USA) with minor modifications to the standard protocol. Wash steps were carried out using freshly prepared 80% ethanol, and DNA was eluted using the elution buffer from the QIAamp PowerFecal DNA Kit (Qiagen, Hilden, Germany).

Sequence processing was performed in QIIME2 [12], where paired-end reads were denoised, merged, and quality filtered using the DADA2 algorithm to generate representative feature sequences and a feature table [13]. Features were clustered into operational taxonomic units (OTUs) at 99% sequence identity, and taxonomic assignment was performed with a pre-trained Naïve Bayes classifier trained on the SILVA 138 database. In total, 202 OTUs were identified across all samples.

**Case studies**

**In vitro aquatic microbiome dataset**

In this case study, we applied MicrobTiSDA to analyze the aquatic microbiome dataset cultured on peptone medium as reported by Fujita *et al*. [8]. The objective was to characterize the temporal dynamics of microbial taxa across eight replicate experiments, to compare interspecies interaction patterns among replicates, and to identify keystone species that drive community-level changes.

The dataset contained a relatively small number of ASVs (28 in total), and therefore no feature filtering was applied. Since the experiment covered a continuous 110-day period without missing time points, interpolation was unnecessary. After performing the MCLR transformation on the raw count data, we fitted natural spline regression models for each ASV within each replicate, excluding ASVs with fewer than 5 nonzero abundance observations. Subsequently, hierarchical clustering was performed on ASVs with similar temporal patterns (correlation < 0.15). Notably, due to substantial compositional differences across replicates, the number of abundance clusters varied among the eight microbial communities, reflecting distinct trajectories (Figure S2). At the individual taxon level, all replicates consistently showed that *Aeromonas* (X_0002) maintained a relatively high and stable abundance over time (Figures S3A–S10A). In addition, in the seven replicates where *Enterobacterales* (X_0010) was detected (Rep.1 – Rep.7), this taxon exhibited a marked increase in abundance after approximately day 70, largely synchronous with the dynamics of *mangenotii* (X_0004) (Figures S3B–S10B). These findings indicate that certain temporal abundance patterns are conserved across replicate aquatic microbial communities.

We further applied *MicrobTiSDA* to infer species interaction networks within each replicate. In this context, species that engage in interactions with at least two other species (*i.e.*, possessing non-zero interaction coefficients with two or more partners) were defined as keystone species. The results revealed highly replicate-specific network topologies, reflecting differences in community composition (Figure S11). For example, more than 10 significant pairwise interactions were detected in Rep.1 and Rep.8, whereas no significant interactions were observed in Rep.6. Moreover, *Aeromonas* (X_0002) exhibited multiple interactions with other ASVs in all replicates except Rep.4 and Rep.6, while *Herbaspirillum* (X_0024) also showed extensive associations across several replicates (Figure S11). Together, these results suggest that *Aeromonas* and *Herbaspirillum* may function as keystone species in regulating community dynamics within peptone-based aquatic microbial communities.

**Infant gut microbiome dataset**

The second case study aimed to investigate the temporal dynamics of the gut microbiota in a pair of dizygotic twins (ID10 and ID11) from day 6 to day 60 after birth, and to identify keystone species potentially driving microbial community changes.

Using *MicrobTiSDA*, we first filtered out OTUs with a total abundance below 5000 and an occurrence frequency of less than 20% samples, resulting in 47 OTUs retained for subsequent analyses. Missing time points between day 6 and day 60 were then interpolated to construct continuous time series. The interpolated data were subjected to MCLR transformation and subsequently used to infer interspecies interactions. The results revealed that ID10 harbored substantially more negative interactions than ID11 (13 interaction pairs vs 5 interaction pairs, Figures S12A,B). This difference may be related to the antibiotic treatment administered to ID10, as previous studies have suggested that antibiotics can enhance competitive interactions within microbial communities [14].

To identify potential keystone species, microbial features engaged in three or more significant interactions were defined as keystone species for the respective individual. Analysis revealed two shared keystone species between the twins: undefined *Bifidobacterium* and undefined *Salmonella*. In addition, ID10 exhibited several unique keystone species, including undefined *Enterobacterales*, undefined *Lachnospiraceae*, undefined *Staphylococcus*, undefined *Veillonella*, and undefined *Clostridium*, whereas ID11 harbored only one unique keystone species, undefined *Niameybacter*. These findings suggest that the gut microbiota of ID10 was more dynamic and sensitive to perturbations: fluctuations in the abundance of multiple keystone species could broadly affect other community members, and the greater number of keystone species implies a higher susceptibility of the community to external disturbances such as *S. agalactiae* infection and subsequent antibiotic treatment.

To further characterize temporal abundance patterns and their similarity between the twins, we fitted natural spline regression models to the filtered OTU abundances, setting the onset (day 22) and termination (day 33) of antibiotic treatment in ID10 as spline knots. Hierarchical clustering (correlation distance < 0.15) was then applied to group the fitted temporal trajectories (Figures S12C,D). The results showed that OTUs in ID10 and ID11 formed 15 and 14 clusters, respectively, reflecting individual-specific temporal dynamics. OTUs annotated to the same taxon generally exhibited highly consistent trajectories within clusters (Figures S15 and S16). Notably, despite ID10’s hospitalization due to *S. agalactiae* infection and subsequent antibiotic therapy during days 22–33, both infants harbored two *Streptococcus*-annotated OTUs (OTU_000011 and OTU_000042) with comparable temporal profiles, characterized by an initial decline followed by recovery. However, we observed a stronger decline of these OTUs in ID10 than in ID11 during the treatment period (days 22–33, Figures S12E,F).

Additionally, to evaluate the robustness of *MicrobTiSDA* in inferring microbial interaction networks, we conducted a systematic sensitivity analysis of key model parameters using this dataset. Specifically, two aspects were examined:

1. Abundance centering method: Prior to interaction inference, abundance data must be centered to reduce the influence of overall abundance levels across microbial features. We compared two centering strategies: mean-based and median based centering. The mean method reflects the central tendency of the entire community, whereas the median method is more robust to outliers and may better capture the baseline of feature abundance. By evaluating both strategies, we aimed to test whether the choice of centering method substantially affects the inferred network structure.
2. MSE threshold: During the stepwise regression procedure of the *LIMITS* algorithm, interaction terms are incorporated only when the prediction error improves beyond a predefined threshold. We examined six threshold levels (10^-1^, 10^-2^, 10^-3^, 10^-4^, 10^-5^, 10^-6^), covering a spectrum from lenient to stringent criteria. Higher thresholds may allow more interaction terms, capturing potentially more complex network structures, whereas lower thresholds yield sparser but potentially more reliable networks by minimizing false-positive associations. Repeating the inference across these thresholds allowed us to assess the stability of results under varying stringency.

Together, these settings yielded 12 parameter configurations (Table S2). For each configuration, *MicrobTiSDA* was applied to the gut microbiome time-series data of two individuals to infer species interaction matrices. We then computed Spearman correlation coefficients between all inferred matrices to quantitatively assess the consistency of results across parameter configurations.

The analysis demonstrated that the inferred networks were highly consistent under all conditions, with all pairwise correlation coefficients exceeding 0.7 (Figure S1). These results indicate that the linear interaction framework implemented in *MicrobTiSDA* is robust to variations in methodological assumptions and provides stable and reliable inference outcomes.

**Gut microbiome dataset of preterm infant with sepsis**

In this case study, we applied *MicrobTiSDA* to a subset of longitudinal gut microbiota data from preterm infants diagnosed with sepsis, aiming to compare the temporal dynamics between infants with *Escherichia coli*-associated sepsis and matched controls. This example highlights the utility of *MicrobTiSDA* for microbiome investigations at the population level.

We first employed a random forest classifier, randomly assigning 70% of samples to the training set and 30% to the testing set. Using all OTUs as input features, the model achieved high predictive accuracy, with an out-of-bag error rate of 0.081 (Figure S15A). Through five iterations of 10-fold cross-validation, we identified the top 10 OTUs with the highest mean decrease accuracy (MDA) as candidate biomarkers distinguishing the two groups (Figure S15D). A random forest model constructed with these 10 OTUs yielded a cross-validation error of approximately 0.9 (Figure S15B). Consistently, NMDS analysis demonstrated that these OTUs effectively discriminated between sepsis and control samples (Figure S15C). Interestingly, the detection frequencies of these OTUs differed markedly between groups (Table S3). OTU_0001 and OTU_0006, which exhibited high prevalence in both groups, were identified as shared biomarkers. In contrast, OTU_0005, OTU_0012, OTU_0013, and OTU_0014 were predominantly detected in controls (> 50%), and thus defined as control-specific biomarkers, whereas OTU_0009, OTU_0015, OTU_0023, and OTU_0031 were enriched in the sepsis group (> 50%), and classified as sepsis-specific biomarkers.

The selected biomarker OTUs were then subjected to MCLR transformation, and for each group, we fitted mixed-effects natural spline regression models with sampling time as a fixed effect and individual ID as a random effect. Based on these models, OTU abundances were predicted over continuous 10-day intervals. Hierarchical clustering (correlation distance < 0.15) was subsequently applied to identify OTUs with similar temporal trajectories. This analysis revealed four OTU clusters in the sepsis group and five clusters in the control group (Figures S15E,F).

Cluster-level examination showed that the two shared biomarkers, OTU_0001 and OTU_0006, each formed a distinct cluster and exhibited consistent temporal dynamics across both groups (Figure S16). By contrast, most group-specific biomarkers displayed relatively stable or fluctuating abundance patterns within their respective groups. Notably, in the sepsis group, OTU_0015 and OTU_0023 – both annotated to the genus *Escherichia–Shigella* – clustered together and showed an increasing trend in abundance over time. This observation suggests that the detection of pathogenic *E. coli* in the bloodstream of septic preterm infants may be linked to extensive colonization of *Escherichia–Shigella* in the gut [15].

**REFERENCES**

1. Faust, Karoline, Leo Lahti, Didier Gonze, Willem M. de Vos, Jeroen Raes. 2015. “Metagenomics meets time series analysis: unraveling microbial community dynamics.” *Current Opinion in Microbiology* 25: 56-66. <https://doi.org/10.1016/j.mib.2015.04.004>

2. Rabbath, Camille Alain, Daniel Corriveau. 2019. “A comparison of piecewise cubic Hermite interpolating polynomials, cubic splines and piecewise linear functions for the approximation of projectile aerodynamics.” *Defence Technology* 15: 741-757. <https://doi.org/10.1016/j.dt.2019.07.016>

3. Yoon, Grace, Irina Gaynanova, Christian L. Müller. 2019. “Microbial networks in SPRING - semi-parametric rank-based correlation and partial correlation estimation for quantitative microbiome data.” *Frontiers in Genetics* 10: 516. <https://doi.org/10.3389/fgene.2019.00516>

4. Hofbauer, J., V. Hutson, W. Jansen. 1987. “Coexistence for systems governed by difference equations of Lotka-Volterra type.” *Journal of Mathematical Biology* 25: 553-570. <https://doi.org/10.1007/bf00276199>

5. Hastie. Trevor J., 2017. “Generalized additive models.” In *Statistical models in S*, 249-307. New York: Routledge.

6. Nidheesh, N, KA Abdul Nazeer, PM Ameer. 2020. “A hierarchical clustering algorithm based on silhouette index for cancer subtype discovery from genomic data.” *Neural Computing and Applications* 32: 11459-11476. <https://doi.org/10.1007/s00521-019-04636-5>

7. Breiman. Leo, 2001. “Random forests.” *Machine learning* 45: 5-32. <https://doi.org/10.1023/A:1010933404324>

8. Fujita, Hiroaki, Masayuki Ushio, Kenta Suzuki, Masato S. Abe, Masato Yamamichi, Koji Iwayama, Alberto Canarini, et al. 2023. “Alternative stable states, nonlinear behavior, and predictability of microbiome dynamics.” *Microbiome* 11: 63. <https://doi.org/10.1186/s40168-023-01474-5>

9. de Muinck, Eric J., Pål Trosvik. 2018. “Individuality and convergence of the infant gut microbiota during the first year of life.” *Nature Communications* 9: 2233. <https://doi.org/10.1038/s41467-018-04641-7>

10. Gilbert, Jack A., Dawn Field, Paul Swift, Simon Thomas, Denise Cummings, Ben Temperton, Karen Weynberg, et al. 2010. “The taxonomic and functional diversity of microbes at a temperate coastal site: a 'multi-omic' study of seasonal and diel temporal variation.” *PLoS One* 5: e15545. <https://doi.org/10.1371/journal.pone.0015545>

11. Caporaso, J. Gregory, Christian L. Lauber, William A. Walters, Donna Berg-Lyons, James Huntley, Noah Fierer, Sarah M. Owens, et al. 2012. “Ultra-high-throughput microbial community analysis on the Illumina HiSeq and MiSeq platforms.” *The ISME Journal* 6: 1621-1624. <https://doi.org/10.1038/ismej.2012.8>

12. Bolyen, Evan, Jai Ram Rideout, Matthew R. Dillon, Nicholas A. Bokulich, Christian C. Abnet, Gabriel A. Al-Ghalith, Harriet Alexander, et al. 2019. “Reproducible, interactive, scalable and extensible microbiome data science using QIIME 2.” *Nature Biotechnology* 37: 852-857. <https://doi.org/10.1038/s41587-019-0209-9>

13. Callahan, Benjamin J., Paul J. McMurdie, Michael J. Rosen, Andrew W. Han, Amy Jo A. Johnson, Susan P. Holmes. 2016. “DADA2: High-resolution sample inference from Illumina amplicon data.” *Nature Methods* 13: 581-583. <https://doi.org/10.1038/nmeth.3869>

14. Ramirez, Jaime, Francisco Guarner, Luis Bustos Fernandez, Aldo Maruy, Vera L. Sdepanian, Henry Cohen. 2020. “Antibiotics as major disruptors of gut microbiota.” *Frontiers in Cellular and Infection Microbiology* 10: 572912. <https://doi.org/10.3389/fcimb.2020.572912>

15. Schwartz, Drew J., Nitan Shalon, Kate Wardenburg, Anna DeVeaux, Meghan A. Wallace, Carla Hall-Moore, I. Malick Ndao, et al. “Gut pathogen colonization precedes bloodstream infection in the neonatal intensive care unit.” *Science Translational Medicine* 15: eadg5562. <https://doi.org/10.1126/scitranslmed.adg5562>

**Figure S1** **Heatmap of Spearman correlation coefficients showing the consistency of inferred interspecies interaction matrices across 12 parameter configurations.** Color intensity represents the strength of correlation, ranging from 0 to 1.

**Figure S2** **The hierarchical clustering results of the OTU temporal patterns for 8 replicate experimental in vitro microbiomes.** The blue dashed line indicates the branch cutting point for clustering based on the correlation distance.

**Figure S3** **Visualizations of clusters of ASVs with similar temporal trends in the Rep.1 cultivation of the in vitro aquatic microbiome.** In total, the temporal patterns of 7 microbial ASVs were clustered into 5 clusters. solid curves indicate continuous OTU temporal patterns predicted by regression models; colors represent different OTUs.

**Figure S4** **Visualizations of clusters of ASVs with similar temporal trends in the Rep.2 cultivation of the in vitro aquatic microbiome.** In total, the temporal patterns of 6 microbial ASVs were clustered into 5 clusters. solid curves indicate continuous OTU temporal patterns predicted by regression models; colors represent different OTUs.

**Figure S5** **Visualizations of clusters of ASVs with similar temporal trends in the Rep.3 cultivation of the in vitro aquatic microbiome.** In total, the temporal patterns of 8 microbial ASVs were clustered into 6 clusters. solid curves indicate continuous OTU temporal patterns predicted by regression models; colors represent different OTUs.

**Figure S6** **Visualizations of clusters of ASVs with similar temporal trends in the Rep.4 cultivation of the in vitro aquatic microbiome.** In total, the temporal patterns of 10 microbial ASVs were clustered into 6 clusters. solid curves indicate continuous OTU temporal patterns predicted by regression models; colors represent different OTUs.

**Figure S7** **Visualizations of clusters of ASVs with similar temporal trends in the Rep.5 cultivation of the in vitro aquatic microbiome.** In total, the temporal patterns of 7 microbial ASVs were clustered into 5 clusters. solid curves indicate continuous OTU temporal patterns predicted by regression models; colors represent different OTUs.

**Figure S8** **Visualizations of clusters of ASVs with similar temporal trends in the Rep.6 cultivation of the in vitro aquatic microbiome.** In total, the temporal patterns of 5 microbial ASVs were clustered into 3 clusters. solid curves indicate continuous OTU temporal patterns predicted by regression models; colors represent different OTUs.

**Figure S9** **Visualizations of clusters of ASVs with similar temporal trends in the Rep.7 cultivation of the in vitro aquatic microbiome.** In total, the temporal patterns of 5 microbial ASVs were clustered into 2 clusters. solid curves indicate continuous OTU temporal patterns predicted by regression models; colors represent different OTUs.

**Figure S10** **Visualizations of clusters of ASVs with similar temporal trends in the Rep.8 cultivation of the in vitro aquatic microbiome.** In total, the temporal patterns of 11 microbial ASVs were clustered into 8 clusters. solid curves indicate continuous OTU temporal patterns predicted by regression models; colors represent different OTUs.

**Figure S11** **Interaction topologies of aquatic microbiomes of eight replicate experiments. Rep.1 to Rep.8 represent replicate experiments 1-8.** Positive interspecies interactions are represented by orange arrows and negative interactions by blue arrows, with arrow width indicating the magnitude of the interaction coefficient. Yellow points represent identified potential keystone species.

**Figure S12** **Results of infant gut microbiome dataset analysis via *MicrobTiSDA*.** (A) and (B) are the internal species interaction inferences of ID10 and ID11, respectively. Positive interspecies interactions are represented by orange arrows and negative interactions by blue arrows, with arrow width indicating the magnitude of the interaction coefficient. Yellow points represent identified potential keystone species. (C) and (D) show the hierarchical clustering dendrograms of the temporal profiles in OTU abundance predicted by the natural spline regression models. The blue dashed line indicates the branch cutting thresholds based on the correlation distance, grouping OTUs with a correlation distance below 0.15 into clusters exhibiting similar temporal patterns. (E) and (F) display the temporal profile clusters of two OTUs annotated to *Streptococcus* for ID10 and ID11. The colors represent OTUs, the points indicate the MCLR-transformed OTU abundances, and the curves represent the trends of the OTUs predicted by regression models, grey shading indicates antibiotic treatment period.

**Figure S13** **Visualizations of clusters of OTUs with similar temporal trends in the ID10’s gut microbiota of the infant gut microbiome dataset.** In total, the temporal patterns of 45 microbial OTUs were clustered into 15 clusters based on a correlation distance threshold of less than 0.15. solid curves indicate continuous OTU temporal patterns predicted by regression models; colors in each cluster represent different OTUs.

**Figure S14 Visualizations of clusters of OTUs with similar temporal trends in the ID11’s gut microbiota of the infant gut microbiome dataset.** In total, the temporal patterns of 43 microbial OTUs were clustered into 14 clusters based on a correlation distance threshold of less than 0.15. solid curves indicate continuous OTU temporal patterns predicted by regression models; colors in each cluster represent different OTUs.

**Figure S15** **Random Forest classification results and temporal pattern clustering of microbial features in the preterm infant sepsis gut microbiome dataset.** (A) Margin scores for the random forest classification model in the training set. (B) Cross-validation curve for the random forest model, the dashed line indicating the optimal number (125) of OTUs based on the minimum error criterion. (C) Non-metric multidimensional scaling (NMDS) demonstrating the separation between predicted sepsis (blue) and control (red) groups. (D) Top 30 biomarkers ranked by Mean Decrease Accuracy from 5 repeated 10-fold cross-validations, the blue bars indicate the top 10 biomarker OTUs selected for mixed-effect natural spline regression models fitting. (E and F) Dendrogram of OTU profiles for the sepsis and control group, respectively, the blue dashed lines indicate branches cutting position.

**Figure S16** **Visualizations of clusters of OTUs with similar temporal trends in the control and sepsis group of the Gut microbiome dataset of preterm infant with sepsis.** The upper panel shows the abundance temporal patterns of 5 OTU clusters in the control group, while the lower panel shows the abundance temporal patterns of 4 OTU clusters in the sepsis group. The curves represent the predicted OTU temporal dynamics based on regression models, the points represent the MCLR-transformed raw counts, and the shapes of the points denote individuals.

**Figure S17** **Schematic figure of the Learning Interactions from MIcrobial Time Series (LIMITS) algorithm.** (A) Data are randomly split into a training set for regression and a test set for evaluating the mean squared error. (B) Stepwise regression is used to determine whether to add species interaction terms to the regression model. (C) Bagging is applied by repeatedly performing stepwise regression on random partitions of the data, each containing half the data points, improving the stability of the fit while maintain the sparsity of the inferred interactions.

**Figure S18** **Sample information for the dataset of 4 septic preterm infants (ID_7, ID_14, ID_19, ID_21) and 4 individually matched control preterm infants (ID_31, ID_37, ID_42, ID_43).** The red cells represent the diagnosis time of sepsis in patients (S_D), the orange cells indicate the matched control individuals (S_C), and the green cells mark the time points of fecal sample collection for each individual. Color blue represents two subjects were clinically matched. The green numbers represent the scaled pseudo days.
